# Short fiber composites from postconsumer textile waste and their suitability in packaging applications

**DOI:** 10.1016/j.heliyon.2025.e42335

**Published:** 2025-01-29

**Authors:** Alva Hjelm, Mikael Skrifvars, Pooria Khalili

**Affiliations:** Swedish Centre for Resource Recovery, Faculty of Textiles, Engineering and Business, University of Borås, SE-50190, Borås, Sweden

**Keywords:** Polymer-matrix composites (PMCs), Compression molding, Recycling, Mechanical properties, Textiles

## Abstract

Large amounts of textile waste are generated annually, and postconsumer blended fiber textiles are particularly challenging to recycle due to difficulties in sorting and limited recycling options, often leading to downcycling or landfilling. This study aimed to create composites from discarded textiles by compressing air-laid nonwoven sheets made from waste viscose and a thermoplastic binder fiber. Ten composite samples, incorporating different types of viscose fibers, were produced using both pre- and postconsumer fabrics to assess the impact of consumer usage, dye presence, and elastane on the composites. A 0.1 wt% NaOH treatment was applied to some postconsumer samples to remove impurities. Mechanical and thermal properties were evaluated using tensile, 3-point bending, impact testing, thermogravimetric analysis, and Fourier transform infrared spectroscopy. The suitability of the composites for packaging applications was also explored, and pilot-scale production demonstrated promising potential for upscaling the process for commercial use.

## 1 Introduction

The rapid expansion of the textile industry over recent decades has led to a significant increase in textile waste generation, with the average European discarding approximately 11 kg of textiles each year [1,2]. The surge in both the purchasing and disposal of textile items has resulted in limited markets for recycling, leading to approximately 60 % of produced clothing being incinerated or landfilled within a year of production [3]. Such waste management practices pose environmental risks, including land pollution, methane emissions, and a substantial loss of valuable materials and business opportunities [4,5]. Recycling these textiles can prevent virgin material production and associated environmental impacts [6,7].

Utilizing recycled textiles as composite reinforcements presents a sustainable solution, as composite materials are already integral in industries such as aerospace, marine, and automotive sectors due to their favorable mechanical properties [[8], [9], [10], [11]]. Natural fiber-reinforced composites have gained interest for being lightweight, biodegradable, and cost-effective [12]. When incorporated as reinforcements, textiles offer unique advantages, such as flexibility, a high specific surface area, and the ability to bear loads axially [13].

However, textile waste often comprises blended fibers, with over a third of garments containing multiple fiber types [14]. For instance, elastane is frequently used to enhance elasticity, while polyester is a common thread material due to its durability [4]. Blended fibers complicate recycling processes, as sorting is essential yet challenging due to various material compositions [15]. Current manual sorting is costly, slow, and labor-intensive [16]. Advanced sorting techniques like Near Infrared Spectroscopy (NIRS) aid in molecular identification, but the analysis may fail to detect embedded fibers, such as elastane within core yarns, which can lead to recycling issues [17,18]. Elastane in particular, commonly found in activewear, poses significant obstacles due to its entanglement in recycling machinery [[19], [20], [21], [22]].

Cellulose, a naturally occurring polymer made up of glucose units in polydisperse linear chains, serves as the foundation for various textile fibers [23]. Cellulosic fibers are categorized based on their origin: natural fibers such as cotton, hemp, jute, and flax and regenerated cellulose fibers like viscose, rayon, lyocell, and modal [24]. Viscose, in particular, holds a notable share in the textile industry, comprising around 10 % of clothing materials by weight [25]. The widespread use of these cellulosic fibers in textiles underscores their potential in composite applications, especially given the structural integrity and versatility they can bring as reinforcement materials.

Postconsumer textiles, which lack detailed content information and may contain impurities from laundering and usage, are another challenge in recycling [[26], [27], [28], [29], [30]]. Factors such as repeated laundering cause polymer degradation, impacting the tensile strength of cellulosic fibers and resulting in low-strength products upon mechanical recycling [[31], [32], [33]]. Dyes and fabric softeners further alter fiber properties, affecting composite strength and quality [[34], [35], [36], [37], [38]].

Fabric production methods like weaving, knitting, and non-woven formation involve interlacing or bonding fibers in various ways, often utilizing mechanical, thermal, or chemical means [[39], [40], [41]]. Given the diversity of textile waste, multiple recycling systems are necessary, with mechanical recycling offering a low-energy and water-efficient solution for a wide range of textiles, despite reducing fiber length [[42], [43], [44], [45]].

Given the complexity and limitations of textile recycling, particularly with postconsumer waste, this study investigates an alternative approach: using postconsumer blended textiles, specifically viscose-based materials, as reinforcement in composites for packaging applications. By comparing pre- and postconsumer textiles, dyed and undyed materials, and mono- and multifiber compositions, this research aims to answer critical questions:

Can postconsumer textile waste serve as a viable material for luxury packaging? Is large-scale production feasible? Are the mechanical properties of recycled composites sufficient to protect and enclose packaged products?

Previous research has demonstrated the use of textile waste in composite materials, though these efforts have primarily focused on industrial and pre-consumer waste sources [[46], [47], [48]]. Unlike industrial waste, postconsumer textile waste presents unique challenges due to its variable content, diverse material properties, and contamination levels, making it more complex to recycle effectively. While some studies have explored the integration of postconsumer textiles in composites [8,49,50], these attempts have predominantly employed thermoset resins as the matrix material, which have inherent limitations such as being inflexible in processing, requiring lengthy curing times, and being difficult to recycle after their initial application. In contrast, this study used thermoplastic binder fibers as the matrix material, offering advantages like shorter processing times, re-meltability, and easier recyclability, making the composites more suitable for circular economy strategies and packaging applications.

Recognizing this gap, the present study pioneers the use of postconsumer blended viscose waste in composites, employing a thermoplastic binder rather than a thermoset resin. By leveraging air-laid nonwoven manufacturing, this study transforms these waste materials into 3D composite forms suitable for packaging applications a unique approach that enhances processing adaptability and material circularity. This novel method offers an alternative pathway for revalorizing complex, blended fiber waste, presenting a viable option for materials that would otherwise face incineration or landfill disposal due to their challenging recycling profile.

## 2 Materials and methods

Table 1 shows the origin of the raw materials used in this study. Each fraction of sample textile was separately shredded. A portion of the postconsumer textiles were subjected to an alkali treatment with 0.1 wt% NaOH after shredding. The thermoplastic binder fibers, which is a type of thermoplastic polymer commonly used in various applications, were mixed with the waste textile fibers and placed in a fiber distributor with rotating axes to evenly distribute the fibers and form an airy mat. The fiber mats were then heated in an oven to partially melt the binder fiber and thermally bind the structure together prior to pressing. A more detailed description of the processing steps has been presented in the section below.

**Table 1:** Material origin and added treatment for each composite sample.

Abbreviation	Full name	Fiber constitution	Fabric composition and material source
**Pr-W**	Preconsumer, White	100 % viscose	White woven fabric from roll.
**Pr-Ww**	Preconsumer, White, washed	100 % viscose	White woven fabric from roll of fabric as in Pr-W, washed ten times in domestic laundry machine and tumble dried after each wash.
**Pr-D**	Preconsumer, Dyed	100 % viscose	Dyed fabric from the same roll of fabric as in Pr-W.
**Pr-Dfs**	Preconsumer, Dyed, fabric softener	100 % viscose	Dyed fabric from the same roll of fabric as in Pr-W.
**Po-P**	Postconsumer, Pure	100 % viscose	Various clothing items in light colors. Only woven.
**Po-Pat**	Postconsumer, Pure alkali treatment	100 % viscose	Various clothing items in light colors. Only woven.
**Po-WM**	Postconsumer, White Mix	90–95 % viscose & 5–10 % elastane	Various clothing items in white. Predominantly knitted.
**Po-WMat**	Postconsumer, White Mix, alkali treatment	90–95 % viscose & 5–10 % elastane	Various clothing items in white. Predominantly knitted.
**Po-DM**	Postconsumer, Dyed Mix	90–95 % viscose & 5–10 % elastane	Various clothing items in white. Predominantly knitted.
**Po-DMat**	Postconsumer, Dyed Mix, alkali treatment	90–95 % viscose & 5–10 % elastane	Various clothing items in white. Predominantly knitted.

### 2.1 Fiber preparations

Five different types of textiles were used to manufacture ten composite samples. White preconsumer fabric was used as a reference to demonstrate the potential differences between the different textile fractions. The white preconsumer reference fabric was used to fabricate four of the ten composite samples, two of which were dyed and two remained white. Postconsumer viscose textile waste was manually sorted into three different categories, pure viscose garments, white viscose garments containing 90–95 % viscose with the residual being elastane and dark colored cellulose garments containing 90–95 % viscose with the residual being elastane.

**Fig. 1:**
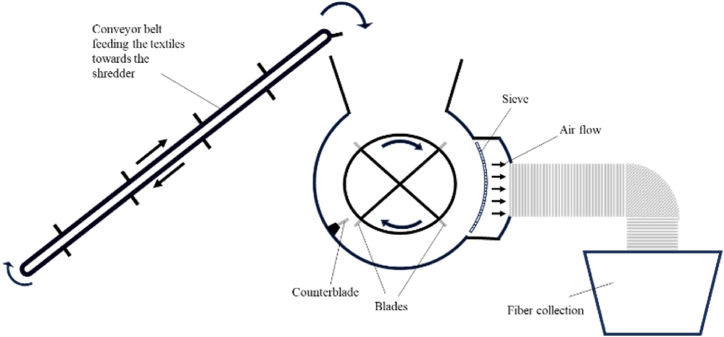
Schematic of the shredding process.

Each fraction was separately placed on a conveyor belt and fed into a shredder which used a rotating blade to cut the textile into a mixture of fibers and small fabric pieces, as shown in Fig. 1. A sieve screen was placed before the outlet to control the size of fibrous material exiting the shredder. The fibrous material was collected and mixed to ensure homogeneity within each batch. Consequently, the shredded material was mixed with a thermoplastic binder fiber, representing the matrix material in the composite. The weight fractions of fibers in the composites were between 5 and 30 wt% for low matrix content and between 30 and 60 wt% for higher matrix content. Before mixing the binder fiber with the viscose material, the fibrous materials were subjected to a large vessel, which utilized compressed air to open the fiber clusters and separate the fibers to ensure even distribution within the air-laid mat. Air-laid nonwoven manufacturing is a dry-laid web formation process in which a fibrous web is created using air as the transportation medium. This process ensures uniform fiber distribution within the mat and avoids the use of water or harsh chemicals, making it highly energy-efficient and environmentally friendly. The method is particularly advantageous for recycling textiles, as it allows for the integration of diverse fiber types and creates lightweight, durable sheets with tailored properties suitable for composite applications. Its versatility extends to applications in hygiene products, insulation, filtration, and packaging materials.

An alkali treatment was performed on one part of each postconsumer textile sample after shredding, namely the fibers from Po-P, Po-WM and Po-DM. This stage was performed to remove dirt and other possible contaminations which the postconsumer garments may have contracted during their usage period. 0.1 wt% of NaOH in an aqueous solution was used to treat the shredded fibrous material at 60 °C for 30 min with a magnetic stirrer as well as manual mixing every couple of minutes (In a previous study [49], it was observed that the chemical treatment of end-of-life textiles with varying concentrations of 2, 4, 6, 8, and 10 wt percent did not enhance the mechanical performance of the respective composites. Moreover, this treatment increased the production costs of the composites. Therefore, a low-concentration alkaline treatment was adopted as a more cost-effective approach, aimed solely at removing surface contamination from the end-of-life textiles and post-consumer textiles.). The fibers were sieved to remove the solution and rinsed with water. Subsequently, the fibers were dried in an oven overnight at 40 °C. The alkali treatment generated the specimens Po-Pat, Po-WMat and Po-DMat. A complete list of sample abbreviations, full name explanations, fiber constitution, fabric composition and material source have been provided in Table 1.

### 2.2 Composite fabrication

**Fig. 2:**
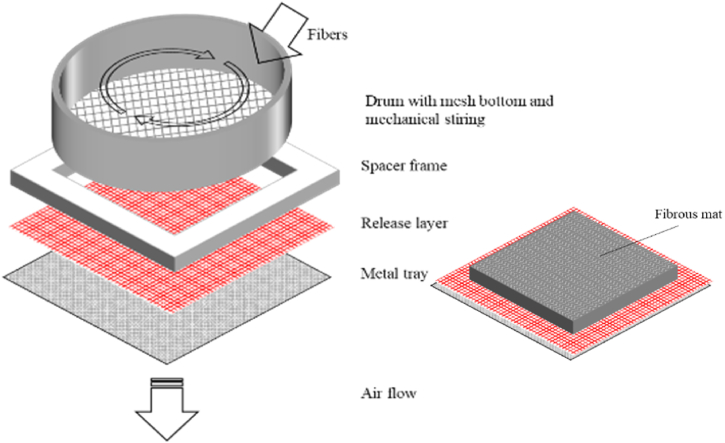
The layers of the fiber mat formation (left) and the layers that placed in the oven for thermal processing and adhesion of the fiber mat (right).

The thermoplastic binder fiber was mixed with the shredded textile in a vessel that used compressed air to mix the fiber blend. When the mix was satisfactory the blend was placed in a fiber distribution machine, or a small air laid nonwoven equipment that evenly distributed the fiber blend through a mesh net and onto a tray with a spacer frame to ensure even nonwoven mats, which is illustrated in the left schematic in Fig. 2. When the spacer frame was filled, the drum and spacer frame were removed, leaving the fiber mat on the metal tray and release layer, as presented on the right-hand side of Fig. 2. The tray, release layer and fiber mat were consequently placed in an oven with circulating air to partially melt the thermoplastic fibers and bind the mat together. The temperature of the oven was set to the melting point of the thermoplastic binder.

The mat was pressed to form a flat composite sheet. Prior to pressing each fiber mat was dried in an oven at 50 °C for minimum of 24 h. The dried mat was placed in a 2 mm spacer frame, sandwiched between two release layers, which was placed in between two metal plates. The beforementioned layers were placed in a 20-tonne bench press between the heated surfaces of the press, without any pressure applied for 60 s. The temperature of the bench press was 5 °C above the binder fibers melting temperature. In the following stage, 3 MPa was applied to the composite for 3 min. After pressing, the composites were placed under 3 KPa for 20 min to cool down and avoid arching. Specimens were laser cut from the composite sheets to use for material characterization. The average thickness and grammage of the resulting composites have been presented in Table 2.

**Table 2:** Average thickness expressed in millimeters of each composite sheet sample together with each standard deviation and grammage expressed in grams per square meters.

	Pr-W	Pr-Ww	Pr-D	Pr-Dfs	Po-P	Po-Pat	Po-WM	Po-WMat	Po-DM	Po-DMat
Mean thickness (mm) ± SD	2.38 0.04	2.41 0.06	2.38 0.06	2.17 0.03	2.43 0.03	2.47 0.05	2.44 0.05	2.44 0.05	2.46 0.04	2.47 0.04
Grammage (g/m^2^)	1500	1550	1500	1200	1300	1250	1650	1400	1450	1500
Density (kg/m^3^)	636	645	627	542	529	508	677	576	597	602

### 2.3 Characterization methods

Thermogravimetric analysis (TGA) was performed with a TGA Q500 from TA instruments. Around 5 mg of fibrous material was rolled into a ball between the palm of gloved hands for each sample. A flow rate of 50 mL/min of nitrogen was used with a heating rate of 10 °C/min until 700 °C. The test was performed in an ambient temperature. Three replications were made for each sample.

A tensile test according to ISO 527–4:2023 was conducted on a Tinius Olsen H10KT tensile testing machine. The test speed was 2 mm/min and a 50 mm extensimeter was used. The specimens were type 1B apart from the thickness, which deviated from the standard. Five replicates were performed for each sample.

To review the fiber pull out behavior from the tensile test a Nikon Eclipse LV100ND microscope was used with the NIS Element BR software to provide a topographical view of the fractured surface. Three areas were scanned on each sample, however only one scan was presented in the study, to represent each composite sample.

A 3-point bending test according to ISO 178:2019 was performed to determine the fracture toughness of the samples. The dimensions of the specimens were 80 mm × 10 mm. The thickness of the samples deviated from the standard. Five replicates were made on each sample.

A Cometech IP-0102-C1I impact testing machine was used to determine the energy absorption of the samples. The testing was performed according to SS-EN ISO 179–2:2020 and the unnotched 80 mm × 10 mm samples were tested in the flatwise direction. As the specimens were cut from a sheet, the thickness was the same as that of the sheet. Five replicates were tested with each sample.

Prior to the tensile, bending and impact test, the specimens were conditioned according to ISO 291:2008 in a climate chamber at 23 °C and 50 % relative humidity.

#### 2.3.1 Processability test

With the knowledge gained from the first section of the study, namely the fabrication and characterization of Pr-W, Pr-Ww, Pr-D, Pr-Dfs, Po-P, Po-Pat, Po-WM, Po-WMat, Po-DM and Po-DMat, the processability in a larger scale production unit was tested to verify upscaling of the process.

Two different fiber mats were produced on a larger scale air-laid production unit, with two different ratios of binder fiber, low (within the range of 5–30 wt%) and high (within the range 30–60 wt%). The different ratios were selected to evaluate the impact of the binder fiber ratio on the tensile properties and on the processability. The air-laid machine operated at the melting temperature of the binder fiber and the fiber mats were pressed to form composite sheets as described in section Composite fabrication, apart from using a 1 mm spacer frame instead of a 2 mm spacer frame. Abbreviations for the composite samples produced in the second section of the project, together with the thickness, grammage and density of the composites were presented in Table 3.

**Table 3:** Abbreviations, thickness, its standard deviation, grammage and density of the composites produced from a larger production unit.

Abbreviation	Average Thickness [mm]	GSM [g/m^2^]	Density [kg/m^3^]
Po-DM(low)	1.28 ± 0.03	1050	820
Po-DM(high)	1.14 ± 0.01	950	827

The parameters for the tensile test were in unity with the standard ISO 527–4:2023 with the sample type 1B, except for the thickness, which have been presented in Table 3. A crosshead speed of 2 mm/min was used together with a 50 mm extensometer. Six replicates were made for each of the two samples and the specimens were conditioned according to ISO 291:2008 prior to testing.

Fourier Transform Infrared Spectroscopy (FTIR) was used to assess potential changes in the chemical bonds of chemically treated samples compared to untreated ones. FTIR analysis was conducted on shredded fiber samples prior to composite manufacturing, using a Nicolet iS10 spectrometer across the spectral range of 4000 cm⁻^1^–400 cm⁻^1^.

## 3 Results

The following sections present the results obtained from the characterization methods performed within the study, namely TGA, FTIR, tensile test, microscopic image analysis, 3-point bending test and impact test. Additionally, a processability trial was conducted on a larger scale production unit to verify the scalability of the process.

### 3.1 Thermogravimetric analysis

**Fig. 3:**
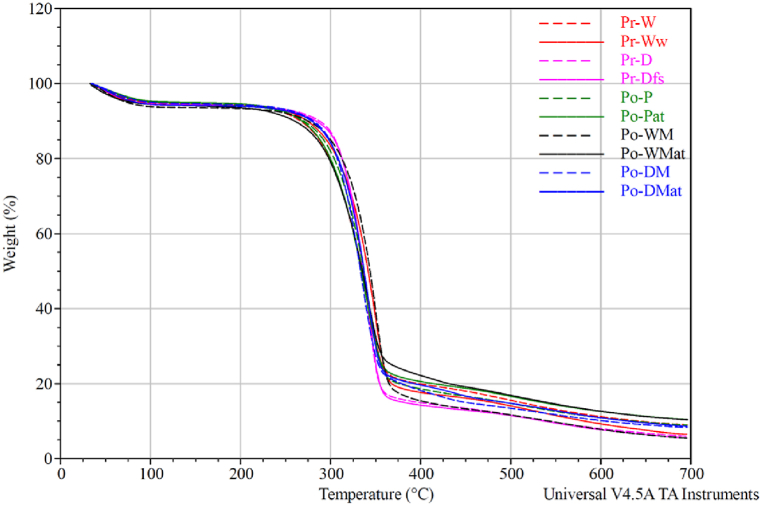
TGA curve of Pr-W, Pr-Ww, Pr-D, Pr-Dfs, Po-P, Po-Pat, Po-WM, Po-WMat, Po-DM and Po-DMat showing weight loss as a function of temperature.

**Fig. 4:**
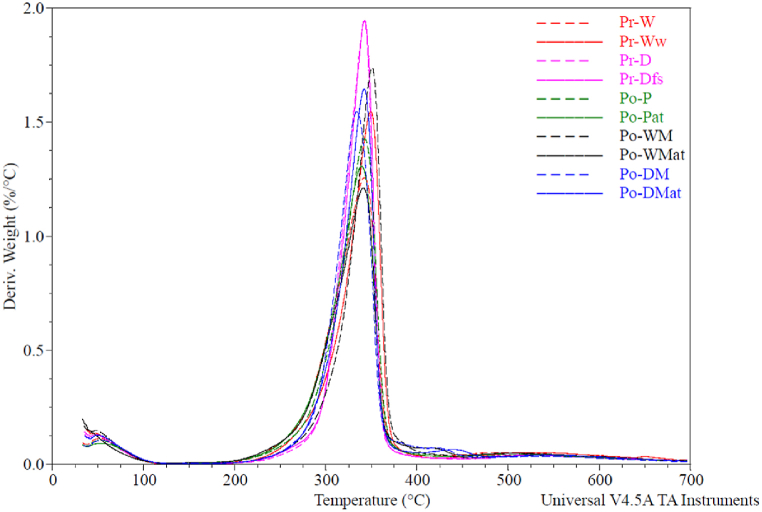
Derivative weight as a function of temperature for Pr-W, Pr-Ww, Pr-D, Pr-Dfs, Po-P, Po-Pat, Po-WM, Po-WMat, Po-DM and Po-DMat.

A thermogravimetric analysis (TGA) was conducted to identify potential differences in contents or degradation behavior within the fiber samples (Fig. 3, Fig. 4). The TGA results were summarized in Table 4, and show the onset- and degradation temperatures expressed in Celsius together with the percentage of moisture, viscose content and residue at 700 °C. The values presented are the average and standard deviations calculated from three replications from each sample type.

**Table 4:** Mean and standard deviation values derived from TGA tests based on a sample size of three.

	Onset temp. (°C)	Degradation temp. (°C)	Moisture content (%)	Viscose content (%)	Residue at 700 °C (%)
**Pr-W**	201.2 ± 2.28	342.5 ± 1.43	5.5 ± 0.34	74.7 ± 0.49	10.9 ± 2.22
**Pr-Ww**	210.8 ± 2.78	349.9 ± 1.16	5.8 ± 0.22	77.4 ± 0.57	8.0 ± 3.33
**Pr-D**	228.5 ± 10.47	342.4 ± 2.12	6.0 ± 0.36	79.8 ± 1.05	6.4 ± 2.74
**Pr-Dfs**	220.2 ± 0.72	342.7 ± 0.06	5.5 ± 0.02	79.7 ± 0.39	5.5 ± 0.19
**Po-P**	210.0 ± 3.21	345.5 ± 2.74	5.4 ± 0.37	74.6 ± 1.47	11.6 ± 2.20
**Po-Pat**	201.0 ± 3.33	340.4 ± 1.19	4.9 ± 0.33	74.0 ± 0.08	10.6 ± 0.07
**Po-WM**	213.8 ± 4.16	349.8 ± 1.33	5.9 ± 0.06	78.1 ± 0.79	6.3 ± 0.96
**Po-WMat**	199.8 ± 5.53	343.0 ± 1.85	5.2 ± 0.47	76.9 ± 2.12	9.7 ± 0.77
**Po-DM**	219.8 ± 2.81	334.9 ± 4.66	5.6 ± 0.56	75.3 ± 1.83	8.2 ± 1.01
**Po-DMat**	207.2 ± 13.91	338.2 ± 3.13	5.7 ± 0.29	73.3 ± 3.25	10.3 ± 2.77

The onset temperature was derived from the derivative thermogravimetric (DTG) curve of each sample, identified as the beginning of the cellulose slope in the DTG curve (Fig. 4). Similarly, the degradation temperature was determined from the peak of the cellulose curve in the DTG, with the average calculated from the three replicates, as presented in Table 4.

The Pr-D sample displayed the highest onset temperature at 228.5 °C, while Po-WMat exhibited the lowest onset temperature at 199.8 °C. These findings contrast with T. Paunikallio, M. Suvanto, and T.T. Pakkanen [51], who reported a higher average onset temperature of 256 °C for viscose fibers in 50 mL/min nitrogen at a 20 °C/min heating rate. Additionally, the alkali-treated samples generally presented lower average onset temperatures compared to their untreated counterparts, indicating a possible impact of the treatment on thermal stability. Specifically, Po-P and Po-Pat had average onset temperatures of 210.0 °C and 201.0 °C, respectively, while Po-WM and Po-WMat had average onset temperatures of 213.8 °C and 199.8 °C. Similarly, for Po-DM and Po-DMat, the onset temperatures were 219.8 °C and 207.2 °C. A two-factor ANOVA, detailed in Table 5, confirmed a statistically significant difference between alkali-treated and untreated groups (p = 0.001) at an alpha level of 0.05. Notably, there was no significant difference in onset temperature across the different postconsumer fiber compositions (p = 0.097), and the lack-of-fit test was also non-significant (p = 0.798), indicating a suitable model fit.

**Table 5:** ANOVA for the significance of alkali treatment on the onset temperature of the postconsumer samples.

Source	DF	Adj SS	Adj MS	F-Value	P-Value
Treatment	1	632.85	632.85	15.76	0.001
Fiber composition	2	222.60	111.30	2.77	0.097
Error	14	562.26	40.16		
Lack-of-Fit	2	20.71	10.35	0.23	0.798
Pure Error	12	541.55	45.13		
Total	17	1417.71			

The results of the alkali treatment differ from findings by Jandas et al. [52], where banana fibers treated with NaOH showed a slight increase in onset temperature compared to untreated fibers. Similarly, Rojo et al. [53] reported an increase in thermal stability for eucalyptus wood cellulose fibers treated with varying NaOH concentrations, attributing this improvement to the removal of wax and impurities with lower decomposition temperatures than cellulose. These impurities likely influence thermal stability by lowering degradation onset for treated samples, which may explain the contrasting results in this study. Additionally, thermal stability in cellulose fibers has been linked to crystallinity and fiber treatments [[53], [54], [55]], which could account for differences observed here.

The mass loss findings in Table 4 also correspond to Paunikallio et al. [51], who found a mass loss of 78.8 % at 600 °C, with testing beginning at 200 °C (without accounting for moisture content, as this study did).

Cellulose degradation typically occurs at approximately 325 °C, as described by Rajan et al. [56]. In this study, degradation temperatures for viscose fiber samples ranged from 334.9 °C (Po-DM) to 349.9 °C (Pr-Ww). Consistent with S. Gaan et al. [57], untreated cellulose exhibited three weight-loss stages upon exposure to elevated temperatures in TGA analysis. The first stage, at around 100 °C, involved moisture evaporation. The second stage, near 360 °C, involved dehydration and carboxylation reactions, releasing gases such as ketones, aldehydes, and ethers, along with char formation, which degraded at around 400 °C in the third stage. This three-stage degradation process, highlighted by S. Gaan et al. [57], aligns with the results observed in the current study, as shown in Fig. 3.

### 3.2 Fourier transform infrared spectroscopy

**Fig. 5:**
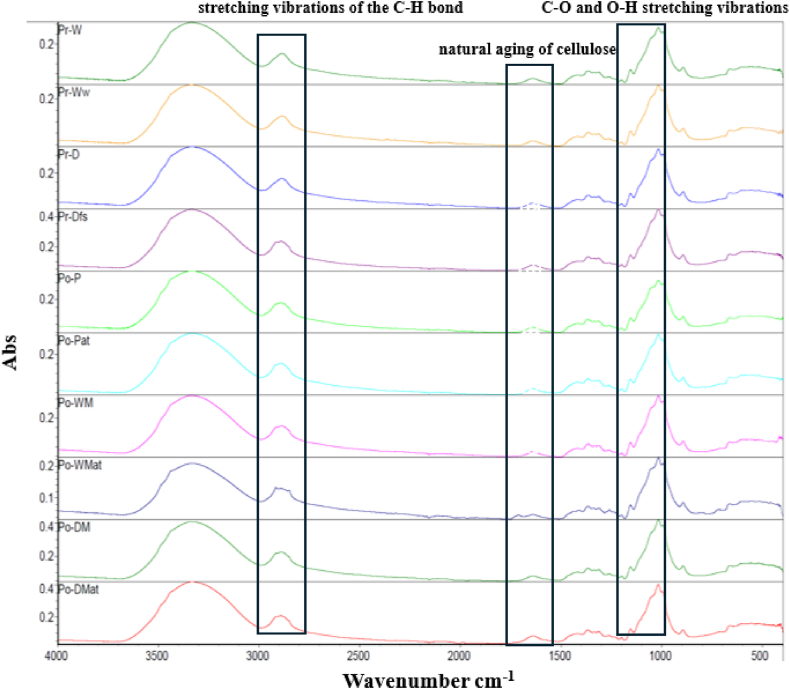
The absorbance spectra obtained from FTIR.

Fourier Transform Infrared Spectroscopy (FTIR) was conducted to assess potential changes in the chemical composition or bonding structure resulting from the treatments applied to the fiber samples. The FTIR spectra, recorded between 4000 cm⁻^1^ and 400 cm⁻^1^, are presented in Fig. 5. Key absorbance peaks were identified and analyzed to understand the effects of chemical treatments on fiber properties.

The absorbance peak at 2923 cm⁻^1^ was attributed to the stretching vibrations of the C-H bond, which indicates the presence of hydrocarbon chains. Additionally, the peaks at 1160 cm⁻^1^ and 1050 cm⁻^1^ were associated with C-O and O-H stretching vibrations, respectively, as reported by Xu, Qi, and Ma [58]. The presence of a small peak around 1640 cm⁻^1^ was attributed to natural aging of cellulose, likely due to partial oxidation of hydroxyl groups to carbonyl groups [59]. This oxidation suggests minor degradation effects potentially linked to fiber storage or use.

Between 1100 cm⁻^1^ and 900 cm⁻^1^, a broad absorbance band was observed, corresponding to various functional groups, including C-C, C-O, and C-O-C stretching vibrations [60]. This region reflects the complex bonding environment of cellulose and other fiber components, indicating the presence of polysaccharide structures inherent in the material.

While no new chemical bonds were detected in the FTIR spectra for treated samples, indicating the absence of major chemical alterations, slight increases in absorbance intensity were noted in Pr-Dfs, Po-DM, and Po-DMat samples. This elevated intensity suggests possible changes in surface chemistry or slight increases in certain functional group concentrations, likely due to the alkali treatment. Alkali treatments can lead to fiber swelling and increased exposure of hydroxyl groups, which may account for the heightened absorbance intensity without forming new bonds.

This lack of bond modification aligns with prior findings, where low-concentration alkali treatments resulted in physical, rather than chemical, changes in fiber morphology. The elevated intensities observed here are consistent with surface-level modifications rather than substantial chemical transformations, supporting the idea that the alkali treatment primarily impacted fiber surface properties, likely enhancing interfacial adhesion in composite applications.

### 3.3 Tensile test results of composites

The results obtained from the tensile test have been presented and summarized in Table 6, Table 7. The ultimate strength of Pr-W was 5.40 MPa on average and 4.98 MPa for Pr-Ww sample, while its dyed counterparts Pr-D and Pr-Dfs had a strength of 4.13 and 3.97 MPa respectively. As the textile reinforcements were of the same origin fabric, the results suggested that the dye impacted the mechanical strength of the composite. Furthermore, the 8 % decrease in tensile strength of Pr-Ww compared to Pr-W indicates that repeated laundering of textile resulted in a decrease in tensile properties as previously reported by A. Palme, A. Idström, L. Nordstierna and H. Brelid [33].

**Table 6:** ANOVA for alkali treatments and fiber compositions impact on the response variable tensile strength for the postconsumer samples.

Source	DF	Adj SS	Adj MS	F-Value	P-Value
Treatment	1	3.4823	3.4823	13.49	0.001
Fiber composition	2	1.8436	0.9218	3.57	0.043
Error	26	6.7114	0.2581		
Lack-of-Fit	2	0.6541	0.3271	1.30	0.292
Pure Error	24	6.0573	0.2524		
Total	29	12.0373

**Table 7:** Average and standard deviation of the ultimate strength, maximum force, elongation at break and E-modulus obtained from the tensile tests for each sample.

	Ultimate strength (MPa)	Maximum Force (N)	Elongation at Break (%)	E-Modulus (MPa)
**Pr-W**	5.40 ± 0.88	128.0 ± 20.5	2.5 ± 0.3	6.45 ± 2.96
**Pr-Ww**	4.98 ± 1.79	120.1 ± 45.3	2.4 ± 0.4	7.16 ± 2.45
**Pr-D**	4.13 ± 0.46	97.0 ± 10.6	2.5 ± 0.6	5.28 ± 2.55
**Pr-Dfs**	3.97 ± 0.81	83.3 ± 16.7	3.2 ± 0.4	3.90 ± 1.10
**Po-P**	2.89 ± 0.14	69.8 ± 3.5	3.5 ± 0.7	2.57 ± 0.74
**Po-Pat**	3.66 ± 0.89	89.6 ± 23.6	3.2 ± 0.7	2.94 ± 0.65
**Po-WM**	3.18 ± 0.37	78.2 ± 8.7	1.9 ± 0.6	4.99 ± 1.66
**Po-WMat**	3.46 ± 0.59	84.4 ± 13.9	2.4 ± 0.7	4.57 ± 1.46
**Po-DM**	2.28 ± 0.22	55.2 ± 5.0	2.4 ± 0.0	3.08 ± 1.53
**Po-DMat**	3.27 ± 0.40	78.8 ± 9.5	2.9 ± 0.5	3.20 ± 1.36

Po-P had an average ultimate strength of 2.89 MPa and the alkali treated counterpart (Po-Pat) showed an ultimate strength of 3.66 MPa, an increment of almost 27 %, which suggested that the alkali treatment effectively increased the samples strength. The increase in tensile strength in Po-WMat was 9 % compared to Po-WM, with ultimate strengths of 3.46 MPa and 3.18 MPa. Po-DMat and Po-DM presented ultimate strengths of 3.27 MPa and 2.28 MPa respectively, which provided an increase of 43 %. A two-factor ANOVA presented in.

Table 6 confirmed the significance of the alkali treatment (p = 0.001). There was also a significant difference between the different postconsumer fiber types in terms of tensile strength (p = 0.043). G.R. Poongodi, N. Sukumar, V. Subramnaiam and Y.C. Radhalakshmi [61] demonstrated that 6 wt% NaOH treated hemp fiber reinforced composites exhibited higher failure strain than their untreated counterpart. The results from this study were consistent with the previously mentioned results, despite the lower concentration of the alkali treatment used in this study. Low concentration NaOH solutions caused the fibers to swell and as a result, the amorphous regions within the fibers were increased [53]. X. Colom and F. Carrillo [62] found that viscose and modal initially possessed lower overall crystallinity after NaOH treatment but there was evidence of recrystallization in alkaline conditions. The authors stated that this was due to the viscose and modal fibers containing cellulose II, amorphous cellulose and transitory regions that were formed in the spinning process, where high molecular chains collapsed. The alkali treatment was assumed to relax the collapsed structures, which in turn promoted reorientation of crystalline structures [62].

The average maximum force ranged from 128.8 N (Pr-W) to 55.2 N (Po-DM). Regarding the elongation at break, it was found to be the highest in Po-P at 3.5 % and lowest for Po-WM at 1.9 %. The E-moduli of the samples ranged from 2.57 MPa (Po-P) to 7.16 MPa (Pr-Ww). Repeated laundering increased the E-modulus by 11 %, as demonstrated by Pr-Ww compared to Pr-W, which is assumed to be due to the particle release induced by washing of the fabric, resulting in a higher longer fiber fraction [63].

**Fig. 6:**
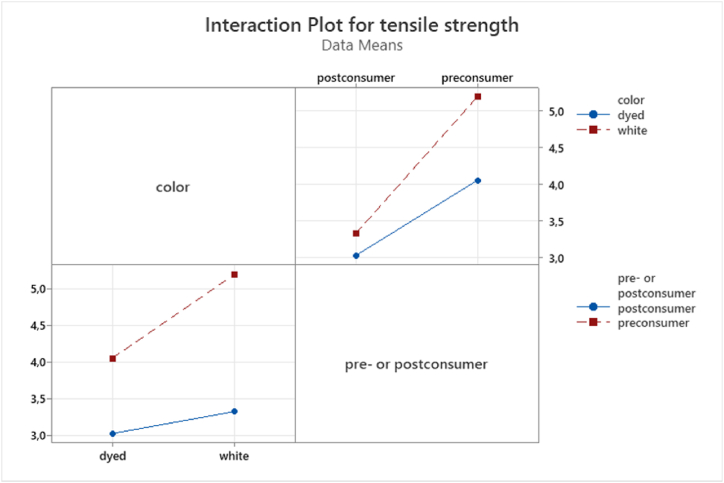
interaction plot for pre- and postconsumer samples that are dyed or undyed.

The results of Pr-W compared to Pr-D were consistent with the results obtained from E. Kumpikaitė, S. Varnaitė-Žuravliova, I. Tautkutė-Stankuvienė and G. Laureckienė [37] and B. Ütebay, P. Çelik and A. Çay [38], where yarns produced from dyed fibers exhibited lower tenacity than greige and undyed fibers. A similar pattern was also observed for the postconsumer samples Po-WM and Po-DM. The interaction plot in Fig. 6 show that pre- or postconsumer fiber origin and dye were significant main effects, however no interaction occurred between the main effects.

As there were certain dimensional differences throughout the samples, as previously presented (in Table 2, Table 3), the specific strength was calculated from the density, to provide a fair comparison amongst the samples. The specific strength was calculated from the ultimate strength converted to the unit N/m^2^. The formula for the specific strength has been presented in the equation below.

$$\sigma_{\text{specific}\;\left[\text{Nm}/\text{kg}\right]}=\frac{\sigma_{\text{ultimate}}\left[N/m^{2}\;\right]}{\rho \;\left[\text{kg}/m^{3}\right]}$$

**Fig. 7:**
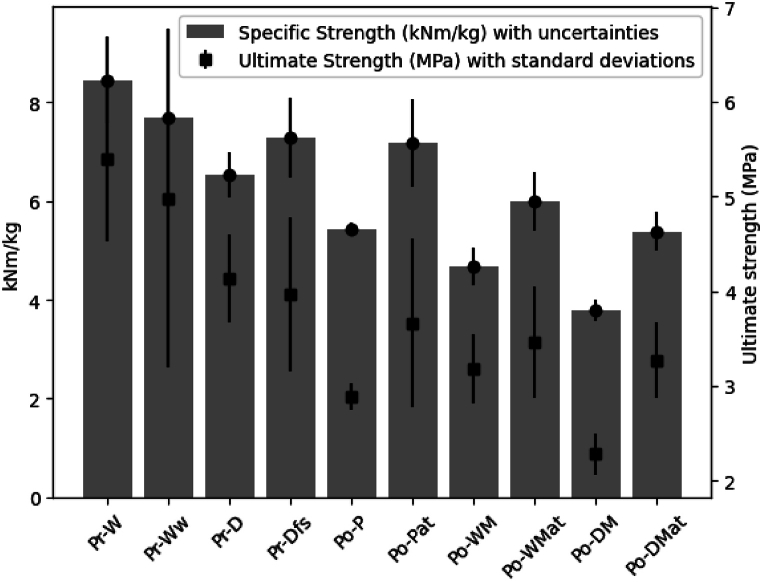
Comparison of Mechanical Properties: specific strength and Ultimate Strength with Uncertainties (specific strength calculated from each sample's ultimate strength divided by its density).

The specific strength of each sample has been presented in Fig. 7. The specific strength and ultimate strength showed similar trends, which indicated that the composite materials were comparable despite the differences in grammage and density. However, Pr-Dfs exhibited higher specific strength than Pr-D, which is a deviation compared to the ultimate strength. Due to being more porous than the other fiber samples as an effect of fabric softener, Pr-Dfs exhibited low grammage and density compared to the other preconsumer samples.

The specimen exhibited elastic behavior, which corresponded to the linear region. Subsequently, the stress-strain curves presented a non-linear region, which corresponded to the initiation of damage in the composites. Finally, before the fracture, the composite exhibited semi-linear evolution as mentioned by J. Fitoussi, M. Bocquet and F. Meraghni [64]. A portion of the specimens exhibited gradual fracture, while in other cases the specimens exhibited an immediate fracture, which is an indication of a more brittle fracture [65].

### 3.4 Microscopic image analysis of the fractured surface of composites

**Fig. 8:**
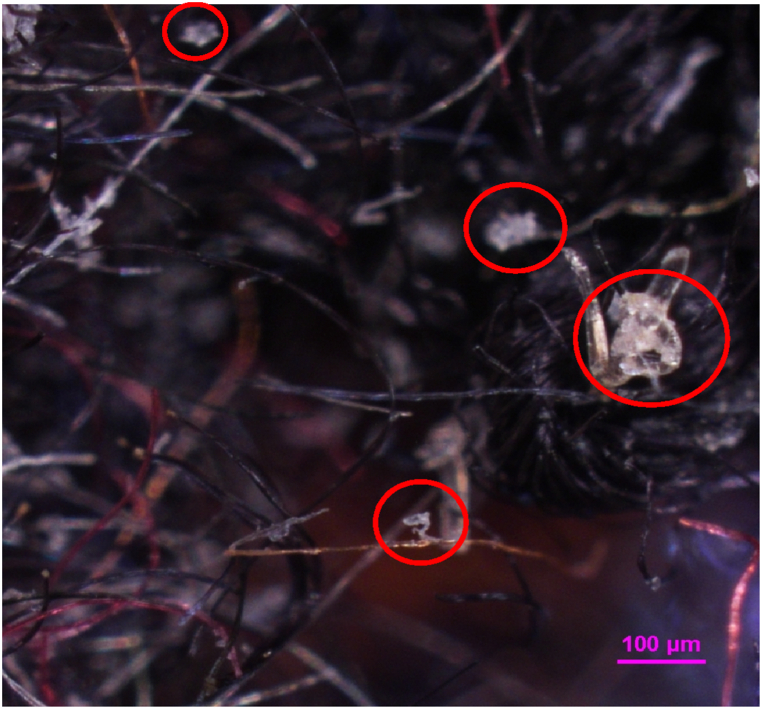
Microscopic image of the cross-sectional area of a Po-DM specimen with the thermoplastic matrix marked in red.

**Fig. 9:**
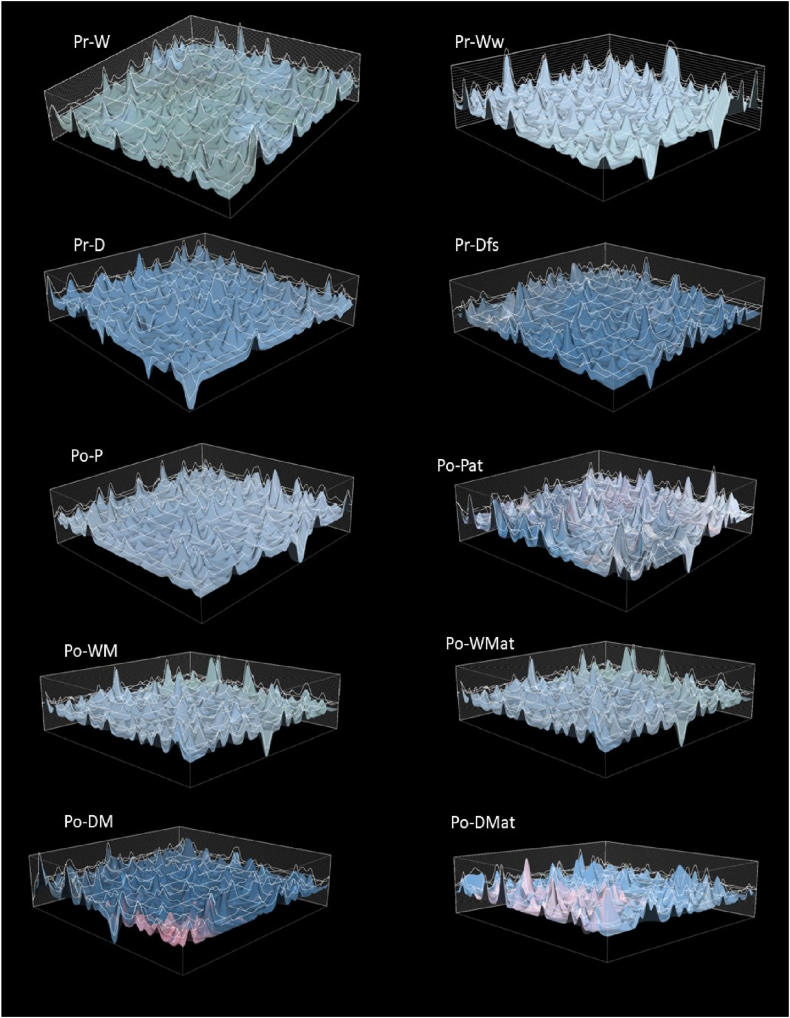
Topographical scans of Pr-W, Pr-Ww, Pr-D, Pr-Dfs, Po-P, Po-Pat, Po-WM, Po-WMat, Po-DM and Po-DMat obtained from microscopic image analysis.

The specimens used for the tensile test were placed under a microscope to display the cross-sectional fracture surface area of the composite. A Nikon Eclipse LV100ND microscope was used together with the NIS Element BR software. As presented in Fig. 8, the binder fibers exhibited shrinkage upon thermal processing and lost their fiber structure. The topographical profiles were displayed in Fig. 9 along with grid to provide visual aid and a scale in the image. The scale of the grid was 100 μm. In terms of failure mechanisms, all specimens showed similar attributes. The surface structure along with the short length of the fiber reinforcements indicated that fiber pull out was the cause of the composite failure [66].

### 3.5 Three-point bending test results of composites

**Fig. 10:**
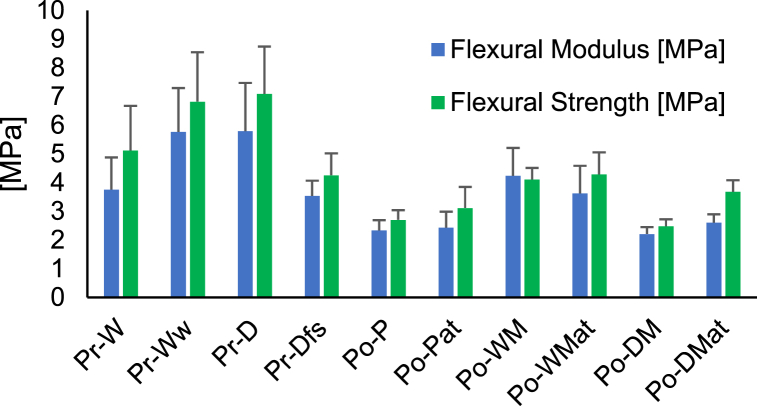
The flexural strength and modulus and their standard deviations of Pr-W, Pr-Ww, Pr-D, Pr-Dfs, Po-P, Po-Pat, Po-WM, Po-WMat, Po-DM and Po-DMat.

The average flexural strength and modulus resulting from the 3-point bending test was summarized in Fig. 10. The undyed Pr-W exhibited lower flexural strength and modulus than the counterpart treated with dye, Pr-D, which indicated that the dye increased the flexural properties. Research conducted by V. Kokol, S.Š. Turk and R. Schneider [67] found that reactive dyes form covalent bonds with the OH-groups in regenerated cellulose fibers, thus impacting the bending rigidity. The preconsumer sample treated with fabric softener after dyeing, Pr-Dfs, exhibited lower flexural properties than Pr-D. It could not be determined whether this effect was caused by the lubricating effect of the fabric softener [36] or the lower grammage compared to the other preconsumer samples as presented in Table 2.

Regarding the postconsumer samples, the flexural modulus was similar for Po-P, Po-Pat, Po-DM and Po-DMat, which indicated that neither the interference of elastane nor alkali treatment impacted this property to a high extent. The flexural strength was however improved by the alkali treatment by 15 %, 4 % and 48 % for Po-Pat, Po, WMat and Po-DMat compared to their untreated counterparts Po-P, Po-WM and Po-DM.

The first part of the curves was seen to illustrate a linear elastic deformation. The maximum displacement of 10 mm was achieved before fracture occurred in the majority of the specimens. A portion of the Pr-Ww and Po-WM samples exhibited failure prior to the 10 mm displacement point and showed tendencies of crack formation on the compressive side of the specimens, which presented as a negative slope after the maximum stress point.

### 3.6 Impact test results of composites

**Fig. 11:**
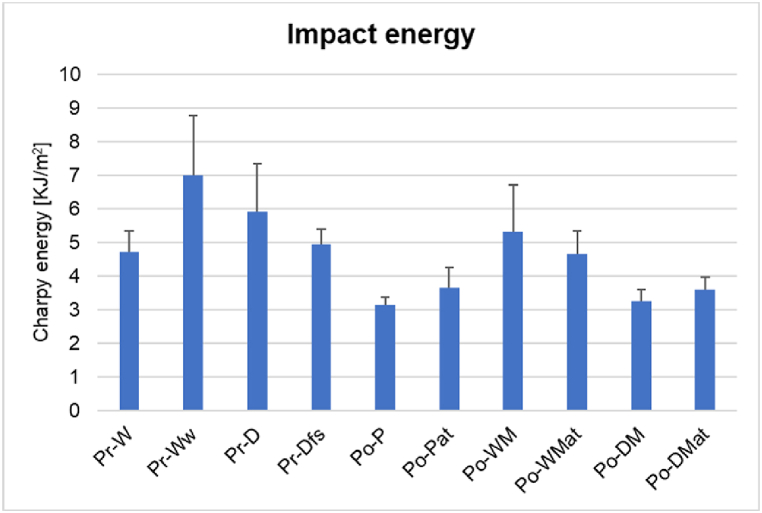
Average Charpy energy and its standard deviation absorbed by the specimens expressed in KJ/m2.

The average Charpy energy absorbed by the composite samples was determined and presented in Fig. 11 together with error bars of standard deviation. Pr-Ww demonstrated an increase of almost 48 % as compared to Pr-W as a result of repeated laundering. The sample which was treated with fabric softener (Pr-Dfs) showed a decrease in impact energy absorption by 17 % as compared to Pr-D, which is assumed to be caused by the fabric softener acting as a lubricant and decreasing the friction between the fibers [36]. The Po-P and Po-DM samples showed similar impact test results, which indicated that the interference of elastane did not negatively affect the impact properties. Po-Pat and Po-DMat exhibited a 16 % and 11 % increase in impact energy absorption, compared to Po-P and Po-DM as a result of the NaOH-treatment. Po-WMat however, presented a decrease in energy absorption by 12 % compared to the untreated sample, Po-WM. It should be noted that the grammage of Po-WM, presented in Table 2 was elevated compared to Po-WMat and the other postconsumer samples, which may be the cause of the deviant result.

Upon impact from the pendulum, the composites underwent plastic deformation, and each specimen assumed a slight V-shape. A tendency of fiber failure was apparent on the backside of the specimens and small creases were visible on the surface where the pendulum had struck the specimen [68].

### 3.7 Processibility trial results

After having completed the first part of the study, namely testing of the ten composite samples Pr-W, Pr-Ww, Pr-D, Pr-Dfs, Po-P, Po-Pat, Po-WM, Po-WMat, Po-DM and Po-DMat, a larger trial run was conducted on a pilot sized air-laid machine with the Po-DM fiber sample. The larger air-laid production unit was used to verify the upscaling of the process established in the first part of the study. Po-DM was selected as it proved most challenging in terms of dye, fiber constitution as well as being from a postconsumer origin. The shredded Po-DM was mixed with two different ratios of binder fiber, low (5–30 wt%) and high (30–60 wt%). Around 2.5 kg of fiber material was processed in the air-laid production unit for each of the specimens named Po-DM(low) and Po-DM(high).

#### 3.7.1 Processability test results

When comparing the processability for Po-DM(low) and Po-DM(high), the shrinkage was more evident in the sample with the higher amount of binder fiber (Po-DM(high)), which caused large holes and cracks in the mat, and raised areas where more material was accumulated. The shrinkage of the binder fiber in Po-DM(high) caused complications in the processing of the mat, which rendered an uneven and unwieldy product. Po-DM(low) also exhibited slight shrinkage upon thermal processing, but to a lesser extent and resulted in a more even and ductile product.

#### 3.7.2 Tensile properties of Po-DM(low) and Po-DM(high)

To determine whether Po-DM(low) and Po-DM(high) possessed suitable mechanical properties for packaging applications, a tensile test was conducted. The tensile test results obtained from Po-DM(low) and Po-DM(high) have been presented in Table 8. Po-DM(high) presented a 21 % increase in ultimate strength and 58 % increase in E-modulus compared to Po-DM(low).

**Table 8:** Tensile test results and their STANDARD DEVIATION values from the samples Po-DM(low) and Po-DM(high).

Parameters	Po-DM(low)	Po-DM(high)
Ultimate strength (MPa)	4.1 ± 0.2	5.2 ± 0.8
Ultimate Force (N)	50.9 ± 2.7	72.3 ± 36.8
Elongation at break (%)	1.9 ± 0.002	1.2 ± 0.007
E-modulus	4.3 ± 0.8	6.8 ± 2.6

### 3.8 Discussion

It has been demonstrated in this study that packaging materials could be produced with postconsumer blended viscose fiber materials. This achievement aligns with the growing demand for sustainable solutions in the circular economy and provides an innovative approach for textile waste management. Mechanical recycling generally results in shortening of textile fibers and low-quality products [1,44]. This fiber degradation occurs due to repeated mechanical stresses that weaken the material structure, limiting its application in high-performance products. Thus, the process presented in this study should not be considered a first option for textile waste. In order to achieve circularity within the textile industry, prioritizing closed-loop systems where fibers retain their original quality is crucial [69]. Chemical recycling can produce virgin quality fibers but has shortcomings in economic and technological aspects [4], making it unsuitable for certain types of waste. This study highlights that mechanical recycling fills a specific gap in textile waste management, particularly for complex and blended fibers that are otherwise non-recyclable through chemical processes [45]. However, it should not be considered a general solution for all textile waste.

The effect of dye has been investigated in this study for both pre- and post-consumer textile composites and a clear trend emerged: samples containing dyed fibers generally exhibited lower tensile strength than their undyed counterpart. These results are consistent with the results obtained from B. Ütebay, P. Çelik and A. Çay [38] and E. Kumpikaitė, S. Varnaitė-Žuravliova, I. Tautkutė-Stankuvienė and G. Laureckienė [37], which showed that fabrics made from yarns that contained dyed cellulose fibers had lower tenacity than those made with greige or gray fibers. This reduction in strength is likely due to the degradation of cellulose during dyeing processes, as exposure to chemicals and heat compromises the fiber's intrinsic properties.

During the processability trial in the air laid machine, complications regarding shrinking and hole formations were evident in both samples (Po-DM(low) and Po-DM(high)), however the shrinkage was more extensive when the fraction of binder fiber was higher, as the binder fiber was the source of this issue. The average tensile strength of Po-DM(high) showed an increase of 27 % as compared to Po-DM(low), however, the tensile properties of Po-DM(low) were more homogenous and predictable than Po-DM(high). This suggests that while higher binder fiber fractions can enhance tensile strength, they come at the cost of structural uniformity and processability. Such trade-offs must be carefully evaluated in an industrial context where material consistency and ease of processing are critical considerations. Additionally, observations from the processing test indicated that using a higher percentage of binder fiber generated bulky mats with major cavities, which could be problematic in the following stages of an eventual production line. Thus, the increased tensile strength of Po-DM(high) compared to Po-DM(low) did not overcome the impracticalities associated with the increased binder fiber fraction.

As the binder in the packaging is thermoplastic, it has the possibility to be remelted and remolded. However, fully recycling the product can be challenging, as the composite material consists of even more different fibers than the blended textile waste. At the very least, the current approach prolongs the material's use-phase, slowing the material loop and postponing the waste management [69]. Additionally, it has the potential to replace virgin material products [6], which contributes directly to the reduction of raw material consumption and overall environmental impact. The discarded textiles are materials which would otherwise be incinerated or landfilled due to their complexity and heterogeneous material composition. Additionally, there are currently few organizations that manage blended postconsumer textiles, indicating that the price for the raw materials would be rather low. This economic aspect makes such processes attractive in regions where collection and sorting infrastructures are established or improving. However, overcoming infrastructural gaps, such as automated sorting technologies, remains critical to scaling up such solutions.

The alkali treatment applied to the postconsumer samples Po-Pat, Po-WMat and Po-DMat increased the tensile strength of all treated samples, by 27 %, 9 % and 43 %. The alkali treatment had little impact on the flexural properties of the samples but increased the impact energy by 16 % for Po-Pat as compared to Po-P and 11 % increase for Po-DMat as compared to Po-DM. However, in Po-WMat the impact absorption was decreased compared to the untreated counterpart Po-WM, by 12 %. This selective improvement in tensile and impact properties suggests that alkali treatment has a varying effect depending on the fiber composition and interaction within the matrix. The reduction in impact energy for Po-WMat may stem from fiber embrittlement caused by the treatment, which requires further investigation. The effect of NaOH treatments on the properties of cellulose fibers have been demonstrated in literature previously by for example D.P. Chattopadhyay [70] who highlighted the possibility to increase the mechanical properties of viscose fibers. However, in a larger scale production setting, alkali treatment would provide further steps in the production line as the alkali treatment also presupposes rinsing and drying. With these additional steps, the simplicity and efficiency of mechanical recycling; one of its main advantages are undermined, as it increases energy and water consumption [45]. Furthermore, further evaluation is required to gain a wider perspective on the potential benefits and limitations of NaOH treatment.

Evidently, the process presented within this study shows potential for viscose and elastane blends but could possibly also be adopted for other complex fiber blends.

The economic feasibility of producing composites from postconsumer viscose fibers is a critical factor in evaluating the practical applicability of the process presented in this study. The use of textile waste as a raw material offers an inherently low-cost input, as these materials are typically either incinerated or landfilled, incurring disposal costs. By repurposing this waste, the production process not only reduces environmental impact but also has the potential to decrease material costs, particularly in regions where textile waste management infrastructure is well-developed.

However, the economic efficiency of the process must be weighed against the additional costs associated with treatments like NaOH, which require rinsing, drying, and energy-intensive steps. These additional processes may undermine the low-cost advantage of using waste materials unless carefully optimized. Future studies could evaluate alternative, cost-effective treatment methods, such as enzymatic or mechanical surface modification, to balance economic and performance benefits.

Scalability also plays a significant role in determining the economic impact. While this study was conducted on a laboratory scale, scaling the process for industrial production could introduce challenges, including the need for automated sorting systems and continuous manufacturing equipment. Nevertheless, the potential market for such composites, particularly in applications like sustainable packaging could justify the initial investment, as demand for eco-friendly alternatives continues to grow. A preliminary cost-benefit analysis, incorporating raw material costs, energy consumption, and potential market pricing for the composites, could provide valuable insights into the process's commercial viability.

Lastly, replacing virgin materials with composites derived from textile waste could offer significant economic advantages by reducing reliance on newly extracted resources and aligning with sustainability-driven incentives, such as government subsidies or certifications for circular economy products. Such market opportunities could offset initial production costs and position these composites as a competitive alternative in sectors where sustainability is prioritized.

## 4 Conclusions

This study demonstrated the feasibility of using postconsumer viscose fabrics as a raw material for composite-based packaging applications, addressing the challenge of recycling complex textile waste that would otherwise be incinerated or landfilled. By evaluating various factors including pre- and postconsumer textile sources, the impact of dyes, laundering, fabric softeners, and elastane content, the study identified specific influences on the mechanical and thermal properties of the resulting composites. Key findings highlight those postconsumer fabrics, particularly those subjected to dyeing or mixed with elastane, generally exhibited reduced tensile properties compared to pre-consumer fabrics. The 0.1 wt% NaOH treatment effectively improved the tensile and flexural strength of postconsumer samples by up to 40 %, notably in the Po-DMat sample compared to untreated Po-DM. However, even with alkali treatment, the mechanical properties of postconsumer composites remained lower than those of preconsumer composites. This suggests that while alkali treatment is beneficial, further process optimization may be needed to enhance the quality of postconsumer composites for demanding applications. For future work, exploring other underutilized blended textile wastes could further expand the range of materials suitable for recycling into composites. Additionally, scaling up the process with a focus on improving binder and fiber integration could provide critical insights into large-scale production viability. Optimization of the production parameters including binder concentration, mat density, and thermal processing conditions will be essential to minimize material waste and meet the quality standards required for commercial packaging applications. Overall, while this process is not a universal solution for textile waste recycling due to limitations in mechanical recycling quality, it provides a promising alternative for specific waste streams and highlights the potential for tailored recycling strategies for different textile types. This study contributes to the advancement of textile recycling technology, supporting circularity in the textile industry through targeted reuse in composite applications.

## CRediT authorship contribution statement

**Alva Hjelm:** Writing – original draft, Visualization, Methodology, Investigation, Formal analysis, Data curation. **Mikael Skrifvars:** Visualization, Supervision, Resources, Project administration. **Pooria Khalili:** Writing – review & editing, Validation, Supervision, Resources, Project administration, Methodology, Formal analysis.

## Ethics statement

The present study, focusing solely on the composite manufacturing for packaging applications, without any potential harm to humans, animals, or other entities, did not require review or approval by an ethics committee.

## Data availability

Data will be made available on request. For requesting data, please write to the corresponding author.

## Declaration of competing interest

The authors declare that they have no known competing financial interests or personal relationships that could have appeared to influence the work reported in this paper.
